# Effect of Gait Training With Non-paretic Knee Immobilization on Lower Limb and Trunk Acceleration in a Post-stroke Hemiparetic Patient: A Case Report

**DOI:** 10.7759/cureus.64193

**Published:** 2024-07-09

**Authors:** Ryosuke Todaka, Tetsu Kajiyama, Naoya Kariu, Masaya Anan

**Affiliations:** 1 Department of Rehabilitation, Beppu Rehabilitation Center, Beppu, JPN; 2 Welfare and Health Science, Oita University, Oita, JPN; 3 Faculty of Welfare and Health Science, Oita University, Oita, JPN

**Keywords:** case report, trunk kinematics, muscle activity, non-paretic knee immobilization, gait training, hemiplegia, stroke

## Abstract

This case report describes a woman in her fifties who experienced a left-sided atherothrombotic cerebral infarction with lesions in the left corona radiata. The patient exhibited motor paralysis of the right upper and lower limbs. After a 10-day acute hospital stay, she was admitted to a rehabilitation facility for an intensive program of physical, occupational, and speech therapy. By day 17 of the onset, she had achieved independence by walking with a cane.

This case was documented to study the effects of gait training with non-paretic knee immobilization on muscle activity and trunk kinematics in post-stroke hemiplegia. Traditional physical therapy was used initially, followed by an intervention phase in which gait training was performed with the non-paretic knee immobilized. This approach was hypothesized to induce beneficial kinematic and muscle activity changes in the paretic limb. The results showed increased muscle activity in the paretic lateral gastrocnemius without compromising trunk stability, suggesting that this method may improve rehabilitation outcomes in similar cases.

## Introduction

After stroke, many patients present with motor paralysis, which is characterized by decreased muscle strength in the paretic lower limb [1], gait asymmetry, and decreased gait speed [2]. Motor function, such as muscle strength and the severity of motor paralysis in the paretic lower limb, is thought to be associated with performance measures such as walking speed [3]. Therefore, efforts to improve the functionality of the paretic lower limb are clinically imperative.

In the quest to improve the functionality of the paretic limb, the importance of use-dependent plasticity is emphasized [4]. In the field of upper limb rehabilitation, the effectiveness of constraint-induced movement therapy has been widely documented [5]. Similar to the upper limb, the efficacy of constraint-induced movement therapy as a proactive strategy for using the paretic limb has been reported in the lower limb [6]. Specific methods include exercises such as gait training, sit-to-stand, and stepping under the constraint of the non-paretic lower limb [7-9]. These methods have been reported to increase the anterior-posterior ground reaction force during forward propulsion of the paretic limb and to increase the single-support time of the paretic lower limb during gait [8]. The change in parameters of the paretic lower limb in the stance phase is attributed to the fact that the knee joint of the non-paretic lower limb is immobilized and the knee joint of the non-paretic lower limb does not bend during the swing phase of the non-paretic lower limb. Lateral flexion of the trunk or extension of the knee joint of the paretic lower limb is required to compensate for the lack of flexion of the knee joint of the non-paretic lower limb during the swing phase [9]. The lateral flexion of the trunk to the paretic side and the increased knee extension angle on the paretic side result in a greater load on the paretic lower limb during the stance phase, which could serve as training to strengthen the paretic lower limb. Increased loading during the stance phase on the paretic side may increase the activity of the ankle plantarflexors, which are antigravity muscles.

However, few studies have demonstrated changes in muscle activity with non-paretic knee immobilization. Gait speed in post-stroke patients is related to the severity of motor paralysis in the paretic lower limb, muscle strength [10], muscle strength in the non-paretic lower limb [11], and trunk stability [10]. In particular, because lower limb strength training combined with gait training after stroke is effective in improving gait speed [12], it is clinically relevant for monitoring changes in muscle activity. In addition, trunk kinematics and concurrent contractions during gait compensate for stability, which merits investigation because of their potential impact on gait stability and joint stiffness [10]. Because gait training with non-paretic knee immobilization often results in compensatory movements such as lateral trunk flexion [9], this lateral trunk flexion may cause excessive trunk instability during gait and lead to co-contraction of the ankle plantar and dorsiflexors to compensate for trunk stability [10]. We hypothesized that gait training with immobilization of the non-paretic knee joint would induce kinematic changes in the stance phase of the paretic lower limb as well as changes in muscle activity and trunk kinematics in the background. This case report examined the effects of gait training with non-paretic knee immobilization on trunk and lower limb kinematic parameters in a post-stroke hemiplegic patient.

## Case presentation

Case introduction

**Figure 1:**
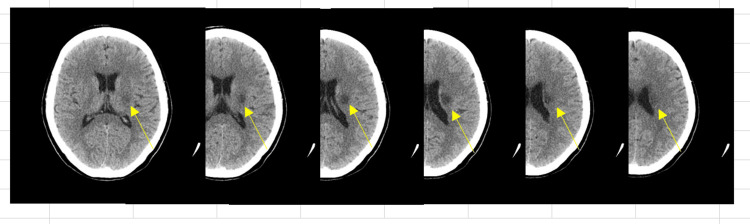
Computed tomography imaging A low-density area from the corona radiata to the basal ganglia was observed.

The patient was a woman in her fifties. She presented with a left-sided atherothrombotic cerebral infarction with lesions in the left corona radiata (Figure 1)

On the day of presentation, the patient took oral aspirin and cilostazol. In addition, intravenous injections of edaravone and argatroban were administered. Initial blood tests revealed a hemoglobin A1c level of 19.9%, indicating the presence of diabetes. She also presented with motor paralysis of the right upper and lower limbs. After a 10-day acute hospital stay for post-stroke treatment, the patient was admitted to our facility for an intensive rehabilitation program that included physical therapy, occupational therapy, and speech-language therapy. As a primary component of physical therapy interventions, activities included gait training, aerobic training using an ergometer, and lower limb strength training. Independence in walking with the use of a cane was achieved by day 17 after the onset, resulting in a functional ambulation category (FAC) score of four. The clinical characteristics of the patient are summarized in Table 1. The patient did not have any cognitive impairments.

**Table 1:** Clinical characteristics of the patient FAC: functional ambulation categories; FMA: Fugl-Meyer assessment; FACT: functional assessment for control of trunk; BBS: Berg balance scale; A1: start of phase A; B1: start of phase B; B2: after two weeks of starting phase B; B3: after four weeks of starting phase B

	Time of admission	A1	B1	B2	B3
FAC (point)	3	4	4	4	4
FMA motor score (upper limb): max = 66 (point)	3	8	12	14	16
FMA motor score (lower limb): max = 34 (point)	28	29	29	29	29
Modified Ashworth scale (elbow/wrist)	1^+ ^/ 1^+^	1^+ ^/ 1^+^	1^+ ^/ 1^+^	1^+ ^/ 1^+^	1^+ ^/ 1^+^
Modified Ashworth scale (hip/knee/ankle)	0 / 0 / 0	0 / 0 / 0	0 / 0 / 0	0 / 0 / 0	0 / 0 / 0
FACT (point)	14	17	20	20	20
BBS (point)	45	46	46	48	52
Comfortable gait speed (m/s)	0.66	0.9	0.87	0.82	0.88

Physical therapy intervention

**Figure 2:**
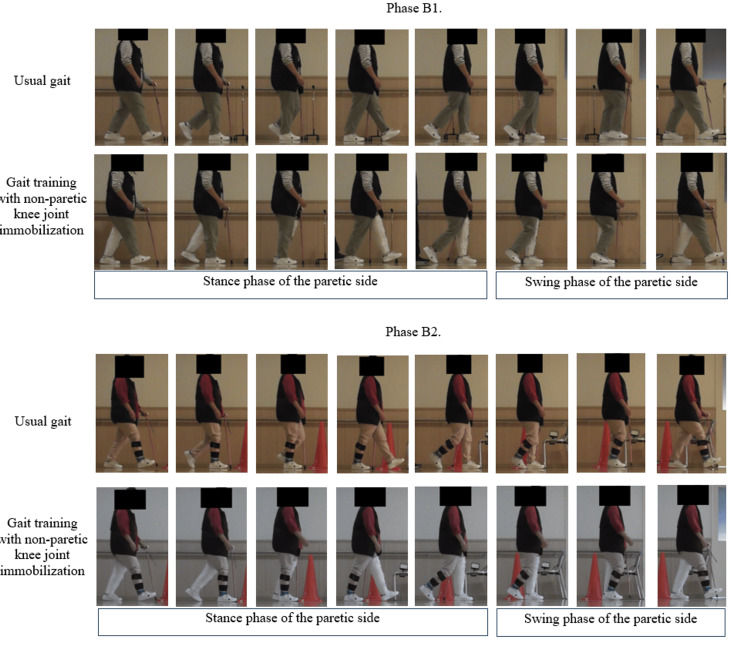
Images of usual gait and training non-paretic knee joint immobilization in phases B1 and B2. These images depict the usual gait and training non-paretic knee joint immobilization in each phase. The first and third images show normal gait, while the second and fourth images show gait training with non-paretic knee joint immobilization. This image is the original work of the authors.

The trial had an AB design. In this case report, the study design was not registered in any database. However, the AB design was used because it was considered optimal for determining the effects of gait training interventions during the post-stroke recovery process. In phase A (days 19-32 after onset), conventional physical therapy consisting of ergometer cycling, gait training, muscle training, and electrical stimulation of the paretic lower limb was performed. During phase B (days 33-65 post-onset), the previously administered conventional physiotherapy gait training was replaced by gait training with the constraint of an immobilized knee joint in the non-paretic lower limb. The non-paretic knee joint was immobilized at 0° of knee extension using a knee brace (Alcare Co., Tokyo, Japan) (Figure 2).

Gait training using a handrail was initiated on the first day of phase B training. Gait training was performed using a cane when the patient was sufficiently acclimatized. The gait speed during gait training was not specified and was a comfortable walking speed. The frequency of implementation was set at five sessions per week, each consisting of five sets of 50 meters. The use of a cane was maintained throughout the experiment. Assessment data were collected at four time points: A1, the beginning of phase A; B1, the beginning of phase B; B2, two weeks into phase B); and B3, four weeks into phase B. Informed consent was obtained from all participants prior to the start of the study. All the procedures were approved by the ethics committee of the Beppu Rehabilitation Center, Beppu, Japan (ethics review number: 27), and conformed to the tenets of the Declaration of Helsinki. This study conforms to all CAse REport (CARE) guidelines and reports the required information accordingly.

Evaluation and data analysis

Physical function assessment of the subjects included the FAC, functional assessment for trunk control (FACT), Berg balance scale (BBS), lower limb motor items of the Fugl-Meyer assessment (FMA), modified Ashworth scale (MAS), and comfortable gait speed.

The study participant walked comfortably on a 10.5-meter walking path with proximal monitoring by a physical therapist to reduce the risk of falls. Gait analysis was performed using surface electromyography (EMG) (Logical Products, Fukuoka, Japan) and a nine-axis wireless motion sensor (Logical Products, Fukuoka, Japan). The EMG electrodes were placed on the lateral gastrocnemius (LG) and anterior tibialis (TA) muscles of the paretic side, according to the Surface EMG for the Non-Invasive Assessment of Muscles Guidelines [13]. The attachment site for the motion sensor was determined to be the spinous process of the third lumbar vertebra (L3), which is consistent with previous studies [14]. In addition, a motion sensor was placed on the heel of the paretic side to identify the gait cycle [15].

The raw EMG signals were bandpass-filtered with cutoff frequencies in the range of 5 Hz to 500 Hz. Rectification smoothing by the root mean square was then performed every 50 ms. The LG muscle activity was quantified by integrating the value obtained by dividing the average amplitude during quiet standing with equal weight distribution on both lower limbs by the stance phase EMG signal. This provided the intensity of muscle activity expressed as a percentage of integrated electromyography (%IEMG). In addition, the co-contraction index (CCI), which represents the degree of simultaneous contraction between the TA and LG muscles on the paretic side, was calculated using the normalized values obtained by dividing the EMG signal during the stance phase by the maximum amplitude during walking, as described in equations (1)-(3).

$(1) CI=\frac{2 I ant}{I total}\times 100$

$(2) I ant =\int_{t1}^{t2} EMG _{TA}(t)dt+\int_{t2}^{t3} EMG_{LG} (t)dt$

$(3) I_{total} = \int_{t1}^{t3}\left [ EMG_{agon}+EMG_{ant} \right ](t)dt$

where Iant is the area of antagonistic muscle activity. The intervals t1 to t2 indicate the period during which TA is smaller than LG, while t2 to t3 indicate the period during which LG has less electromyographic activity than TA. Furthermore, Itotal corresponds to the integrated activity of the TA and LG muscles during gait.

From the angular velocity and acceleration signals obtained from the motion sensor, we estimated the stance time ratio during walking and derived walking indices from trunk acceleration. The stance time ratio was determined from the angular velocity signal attached to the paretic heel to identify initial contact and toe-off [15]. Subsequently, single-leg support time was calculated relative to the period of one gait cycle. The acceleration signal was noise processed with a 20 Hz low-pass filter, and then the acceleration root mean square (RMS) and step symmetry (SS) were calculated. Acceleration RMS describes the average amplitude of acceleration during gait at L3 and serves as an index to assess trunk instability [16]. To examine trunk sway unaffected by gait speed, we normalized it by the square of the gait speed [10] and calculated it for each axis: mediolateral, vertical, and anteroposterior (acceleration RMSx, acceleration RMSy, and acceleration RMSz). The SS was calculated by determining the autocorrelation (AC) of the steps derived from the acceleration at L3 [14]. Calculations were performed for each axis: mediolateral, vertical, and anteroposterior (SSx, SSy, and SSz). The SS showed a higher degree of symmetry, approaching 1 on the vertical and anteroposterior axes. The mediolateral axis is symmetric as it approaches -1. All parameters provided stable data for 10 consecutive gaits.

The changes in paretic lower limb muscle activity, stance time ratio, and trunk acceleration during phases A and B were calculated by calculating the slope from the values at four time points, including phases A and B, and removing the trend. This trend was removed to reduce the effect of spontaneous recovery from a stroke event.

Results of the intervention

Clinical Evaluation

There were no changes in the FAC, comfortable gait speed, FMA-LE score, or MAS throughout the intervention period. The FACT initially improved, whereas BBS improved during phase B (Table 1).

The Course of EMG in the Paretic Lower Limb

**Figure 3:**
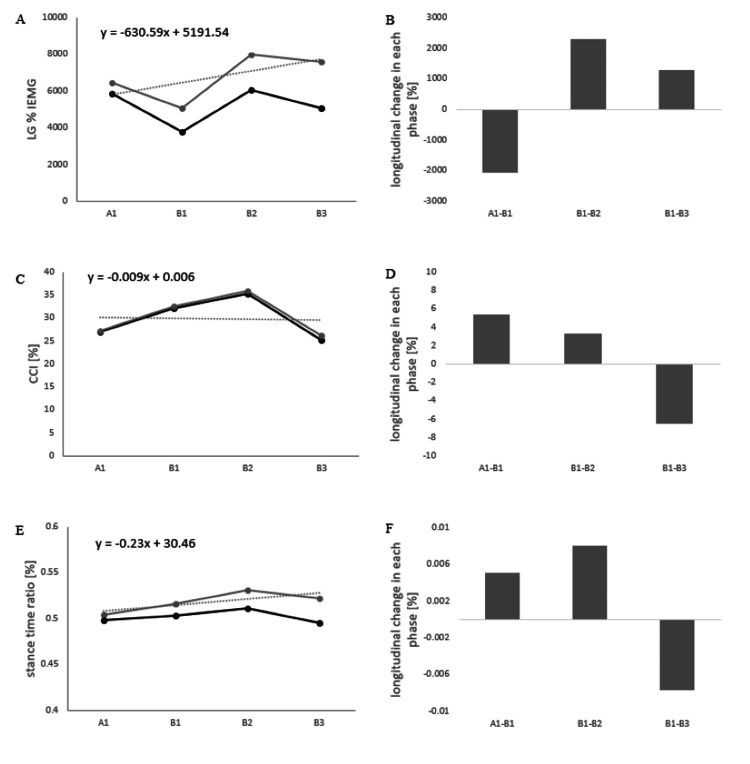
Progress of each parameter and the amount of change in each phase Images A, C, and E show the muscle activity of the paretic limb and the stance time ratio of the paretic side at four time points in phases A1 to B3. The gray lines show the raw data, the black lines indicate the detrended data, and the dashed line indicates the slope of the raw data. Images B, D, and F show the changes in each parameter every two to four weeks from phase A1 to phase B3. LG: lateral gastrocnemius muscle; TA: tibialis anterior; IEMG: integral electromyography; CCI: co-contraction index; A1: start of phase A; B1: start of phase B; B2: after two weeks of starting phase B; B3: after four weeks of starting phase B

**Figure 4:**
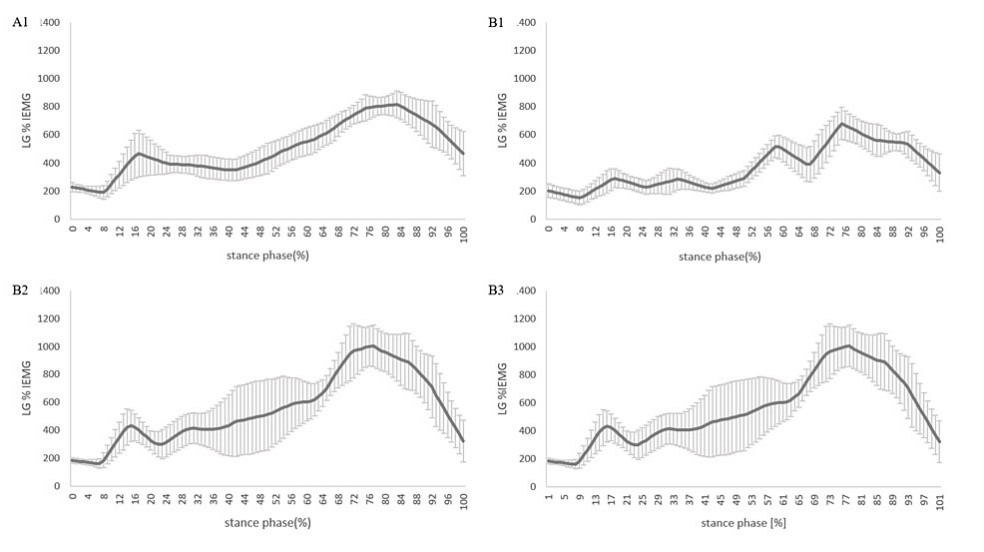
Intensity of muscle activity expressed as a percentage of the integrated electromyography of the LG during the stance phase of gait LG: lateral gastrocnemius muscle; %IEMG: % integral electromyography; A1: start of phase A; B1: start of phase B; B2: after two weeks of starting phase B; B3: after four weeks of starting phase B

The %IEMG values for the A1 phase were 6467.2%, for the B1 phase 5042.9%, 7967.6% for the B2 phase, and 7594.3% for the B3 phase, respectively (Figure 3A and Figure 4). The CCI values were 27.0% in the A1 phase, 32.1% in the B1 phase, 35.2% in the B2 phase, and 25.2% in the B3 phase (Figure 3C). Changes in %IEMG and CCI were calculated every two to four weeks from phase A1 to phase B3 (Figures 3B, 3D). Positive values indicate that the value in each phase was higher than in the previous phase. The %IEMG of LG was -2054.9 in phases A1 to B1, 2294.1 in phases B1 to B2, and 1290.21 in phases B1 to B3. The CCI was 5.3 in phases A1 to B1, 3.31 in phases B1 to B2, and -6.5 in phases B1 to B3.

The Course of the Stance Time Ratio

The stance time ratios for phases A1 phase were 50.5%, B1 phase was 51.6%, B2 phase was 53.2%, and B3 phase was 52.2% (Figure 3E). Changes in stance time ratios were calculated every two to four weeks from phases A1 to B3 (Figure 3F). The stance time ratio was 0.01 in phases A1 to B1, 0.01 in phases B1 to B2, and -0.01 in phases B1 to B3.

The Course of Trunk Acceleration

**Figure 5:**
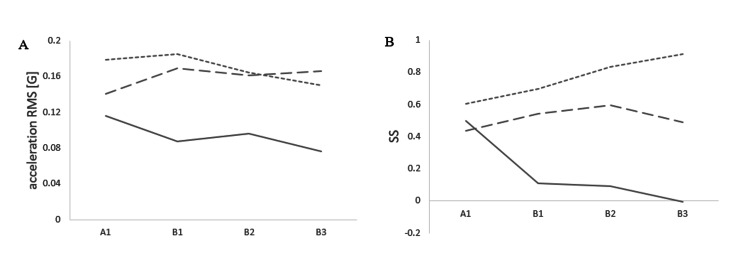
Progress of indices calculated from trunk acceleration The acceleration RMS and SS from trunk acceleration are shown at four time points in phases A1 to B3. Solid lines indicate the mediolateral axis (x), dotted lines indicate the vertical axis (y), and dashed lines indicate the anteroposterior axis (z). RMS: acceleration root mean square; SS: step symmetry; x: mediolateral axis; y: vertical axis; z: anteroposterior axis, A1: start of phase A; B1: start of phase B; B2: after two weeks of starting phase B; B3: after four weeks of starting phase B

The acceleration RMSx (G) values for the A1 phase were 0.12, the B1 phase was 0.09, the B2 phase was 0.10, and the B3 phase was 0.08. The acceleration RMSy (G) values for the A1 phase were 0.18, the B1 phase was 0.18, the B2 phase was 0.16, and the B3 phase was 0.15. The acceleration RMSz (G) values for the A1 phase were 0.14, the B1 phase was 0.17, the B2 phase was 0.16, and the B3 phase was 0.17 (Figure 5A and Table 2). The SSx for the A1 phase was 0.50, the B1 phase was 0.11, the B2 phase was 0.09, and the B3 phase was 0.00. The SSy for the A1 phase was 0.60, the B1 phase was 0.70, the B2 phase was 0.83, and the B3 phase was 0.91. The SSz for the A1 phase was 0.44, the B1 phase was 0.54, the B2 phase was 0.60, and the B3 phase was 0.49 (Figure 5B and Table 2). Changes in acceleration RMS and SS were calculated every two to four weeks from phase A1 to B3 (Table 2). The acceleration RMS (x/y/z) was -0.02 / 0.02 / 0.02 in phases A1 to B1, 0.02 / -0.01 / -0.02 in phases B1 to B2, and 0.01 / -0.01 / -0.02 in phases B1 to B3. The SS (x/y/z) was -0.24 / -0.01 / 0.09 in phases A1 to B1, 0.13 / 0.03 / 0.03 in phases B1 to B2, and 0.19 / 0.00 / -0.10 in phases B1 to B3.

**Table 2:** Progress of indices calculated from trunk acceleration during gait and the amount of change in each phase acceleration RMS: acceleration root mean square; SS: step symmetry; x: mediolateral axis; y: vertical axis; z: anteroposterior axis; A1: start of phase A; B1: start of phase B; B2: after two weeks of starting phase B; B3: after four weeks of starting phase B

	Raw data				After trend removal				Longitudinal change		
	A1	B1	B2	B3	A1	B1	B2	B3	A1 - B1	B1 - B2	B1 - B3
Acceleration RMSx	0.12	0.09	0.10	0.08	0.13	0.11	0.13	0.12	-0.02	0.02	0.01
Acceleration RMSy	0.18	0.18	0.16	0.15	0.19	0.21	0.20	0.19	0.02	-0.01	-0.01
Acceleration RMSz	0.14	0.17	0.16	0.17	0.13	0.16	0.14	0.14	0.02	-0.02	-0.02
SSx	0.50	0.11	0.09	0.00	0.65	0.42	0.55	0.61	-0.24	0.13	0.19
SSy	0.60	0.70	0.83	0.91	0.50	0.49	0.51	0.49	-0.01	0.03	0.00
SSz	0.44	0.54	0.60	0.49	0.41	0.50	0.53	0.40	0.09	0.03	-0.10

## Discussion

This study longitudinally evaluated the effects of gait training with non-paretic knee immobilization on stroke survivors with mild motor paralysis. Gait training with non-paretic knee immobilization showed an increase in muscular activity in the LG during the single-leg support phase. In addition, no changes in gait speed or stance time ratio were observed. Furthermore, this intervention had little or no effect on co-contraction of the paretic lower limb muscles, trunk instability, or trunk symmetry.

A peculiar aspect of this intervention is the increase in activity of the paretic lateral LG muscle during phase B. In the context of restricting the use of the non-paretic limb by applying a prosthesis to the non-paretic limb, an increase in the forward propulsive force of the paretic limb has been documented [8]. In this case, as observed in previous research, the imposition of restrictions on the use of the non-paretic lower limb resulted in an increase in the activity of the LG, which is responsible for forward propulsion. A greater weight shift to the paretic lower limb is required to overcome swing limitations on the non-paretic side due to non-paretic knee immobilization [17]. The shift of weight to the paretic limb may have shifted the proportion of weight support previously distributed between the cane and the paretic limb to the paretic limb, resulting in greater weight support and forward propulsion of the body by the paretic limb, leading to an increase in LG integral values.

In individuals with post-stroke hemiparesis, a reduction in the contribution of the paretic lower limb to forward propulsion during gait is often observed [18], indicating reduced use. This case presented with relatively mild motor paralysis and a reduced stance phase compared to normal conditions. Previous studies have reported that restricting the use of the non-paretic lower limb is associated with an increase in the stance phase ratio [8]. This intervention was expected to increase the percentage of the stance time ratio, but the change was only a few percent compared to the beginning of the intervention. The stance time of the paretic lower limb is influenced by the movement pattern of the lower limb during the stance phase. It has been reported that when the knee undergoes excessive flexion or extension during the stance phase, the stance time is shortened [19]. In a clinical context, gait with the non-paretic knee joint immobilized may manifest the occurrence of knee hyperextension. In this intervention, although the obvious manifestation of knee hyperextension was not observed during gait observation, a more in-depth study of the movement patterns of the paretic lower limb is necessary to achieve an extension of the stance-time ratio. In addition, gait speed did not improve, likely because of the insufficient extension of the stance time ratio. For the trunk, the acceleration RMS showed no obvious changes, and the SS also showed no obvious changes, except for the mediolateral axis. The mediolateral axis of the SS is considered highly symmetric when approaching -1 [14]. Therefore, gait asymmetry was initially observed in both mediolateral axes; however, it decreased over the course of the intervention period. Trunk acceleration deviation is affected by lower limb motor paralysis [20]. Because the motor paralysis, in this case, was mild and there was no impairment of trunk function, it is possible that gait with a non-paretic knee immobilization did not increase trunk sway or asymmetry in trunk acceleration, allowing walking without difficulty.

The limitations of the study include limited EMG measurements of the two muscles and a lack of information on sustained effects. Future research should include a larger sample size and longitudinal verification. In addition, it is possible that co-contraction occurred in the trunk muscle group to maintain posture during gait, but this has not been demonstrated. The lack of a substantial change in trunk acceleration may have contributed to some compensatory action on the trunk muscles, and further verification is needed.

## Conclusions

This intervention aimed to increase the use of the paretic lower limb during gait, and gait training was performed with non-paretic knee immobilization in a post-stroke hemiplegic patient. This resulted in increased muscle activity in the paretic LG. Furthermore, the effect on trunk acceleration was minimal, and compensatory movements such as simultaneous contraction of the paretic lower limb were not observed. This finding suggests that the motor strategy for gait in the paretic lower limb may be altered by limiting the use of the non-paretic lower limb. Further research is needed to consolidate these findings and explore broader applications for post-stroke rehabilitation.
